# Bioinformatic Prediction of Gene Functions Regulated by Quorum Sensing in the Bioleaching Bacterium *Acidithiobacillus ferrooxidans*

**DOI:** 10.3390/ijms140816901

**Published:** 2013-08-16

**Authors:** Alvaro Banderas, Nicolas Guiliani

**Affiliations:** Laboratory of Bacterial Communication, Department of Biology, Faculty of Sciences, University of Chile, Santiago 780-0024, Chile

**Keywords:** *Acidithiobacillus ferrooxidans*, AfeR, biofilm, biomining, quorum sensing, MEME

## Abstract

The biomining bacterium *Acidithiobacillus ferrooxidans* oxidizes sulfide ores and promotes metal solubilization. The efficiency of this process depends on the attachment of cells to surfaces, a process regulated by quorum sensing (QS) cell-to-cell signalling in many Gram-negative bacteria. *At. ferrooxidans* has a functional QS system and the presence of AHLs enhances its attachment to pyrite. However, direct targets of the QS transcription factor AfeR remain unknown. In this study, a bioinformatic approach was used to infer possible AfeR direct targets based on the particular palindromic features of the AfeR binding site. A set of Hidden Markov Models designed to maintain palindromic regions and vary non-palindromic regions was used to screen for putative binding sites. By annotating the context of each predicted binding site (PBS), we classified them according to their positional coherence relative to other putative genomic structures such as start codons, RNA polymerase promoter elements and intergenic regions. We further used the Multiple EM for Motif Elicitation algorithm (MEME) to further filter out low homology PBSs. In summary, 75 target-genes were identified, 34 of which have a higher confidence level. Among the identified genes, we found *afeR* itself, *zwf*, genes encoding glycosyltransferase activities, metallo-beta lactamases, and active transport-related proteins. Glycosyltransferases and Zwf (Glucose 6-phosphate-1-dehydrogenase) might be directly involved in polysaccharide biosynthesis and attachment to minerals by *At. ferrooxidans* cells during the bioleaching process.

## 1. Introduction

*Acidithiobacillus ferrooxidans* is an acidophilic Gram-negative autotrophic γ-proteobacterium that obtains its energy through the oxidation of ferrous iron and reduced inorganic sulfur compounds. *At. ferrooxidans*, along with other bacterial, archeal and eukaryotic microorganisms forms structured biofilm communities in metal-rich low pH environments, where chemolithotrophic metabolism is the primary form of energy acquisition [1]. The bioleaching process that takes place in such systems is employed in biomining biotechnology [2]. Several studies reveal that bioleaching efficiency is improved by biofilm formation on minerals [3,4]. However, to date the molecular mechanisms involved in the regulation of biofilm formation by bioleaching microorganisms are still unknown. The characterization of the molecular mechanisms that take place when *At. ferrooxidans* interacts with its energetic substrate is an important step towards rational design strategies to improve bioleaching efficiency [3].

Bacterial cell-cell communication is based on the regulation of gene expression of interacting bacteria through the exchange of low-weight organic compounds or small peptides that function as signals. Signaling mechanisms have a prominent role in the emergence of cooperative behaviors in bacterial populations and communities [5]. Quorum sensing (QS) is a bacterial cell-cell signaling system that can work as a gene expression coordination mechanism [6]. *N*-Acyl homoserine lactone (AHL) mediated QS (AHL-QS) is the most intensively studied system. It has been characterized in great detail in Gram-negative bacteria. Molecular components of this system include LuxR-family transcriptional regulators, 18–20 nucleotide *cis*-acting elements called *lux-boxes*, AHLs and LuxI-family AHL synthases. LuxR-family proteins are AHL-responsive global transcriptional regulators that activate or repress transcription upon interaction with *lux-boxes* [7]. QS regulates expression of genes related to diverse phenotypes in many bacteria including biofilm formation, swarming motility, bioluminescence and conjugation [8]. AHL molecules have also been implicated in phenotypes related to surface adhesion either directly as biosurfactants [9] or as a signal for transcriptional regulation of adhesion determinants [10,11].

*At. ferrooxidans* strain ATCC 23270 possesses a functional AHL-based QS system [12,13]. This bacterium has one pair of *luxI*/*luxR* homologs termed *afeI*/*afeR*. Here, the QS locus comprises the divergently organized *afeI* and *afeR* genes and a third gene (*orf3*) located in the intergenic region between these two and with the same orientation as *afeI.* The protein product of *orf3* is conserved among some bacterial species but its function is unknown. The synthase AfeI drives the synthesis of at least nine different AHL molecules with medium or large acyl side chains [7] and AfeR is the transcriptional regulator responsible for sensing the AHL concentration. To date, the only known target of the AfeR protein is the palindromic sequence upstream of *afeI.* This was demonstrated by using a heterologous biosensor construct in *E. coli* where AfeR promotes AHL-dependent transcription of *afeI* [13]. On the other hand, the *afe-box* regulatory element located upstream of *afeI* is an 18–20 bp palindromic sequence with nearly perfect dyad-symmetry. It overlaps the -35 region of a sigma 70 promoter consensus sequence, as it is in many other bacteria. However, the *afe-box* has the particularity of being the central fraction of a larger 30 bp palindrome with which it shares the same center of symmetry. Moreover, each half of the 30-bp sequence shows palindromy. Hence, two alternative centers of symmetry can be found in the total sequence [12].

Recently, it has been described that AHL molecules improve the attachment of *At. ferrooxidans* cells to solid energetic substrates such as sulfur and pyrite [14]. As a step towards identifying the molecular mechanism involved in biofilm formation in *At. ferrooxidans*, we have used a bioinformatic strategy to identify genes whose expression might be regulated by AfeR, as it has been described for other transcriptional regulators including CepR, a LuxR-family protein [15,16]. Based on the modular arrangement of the *afeI* operator we constructed Hidden Markov Models (HMMs) to screen for putative binding sites for AfeR in the *At. ferrooxidans* ATCC 23270 genome. Results revealed that at least 75 genes corresponding to 2.6% of the identified ORFs from *At. ferrooxidans* could be direct targets for AfeR.

## 2. Results and Discussion

### 2.1. Construction of the *afe-box* HMMs

**Figure 1:**
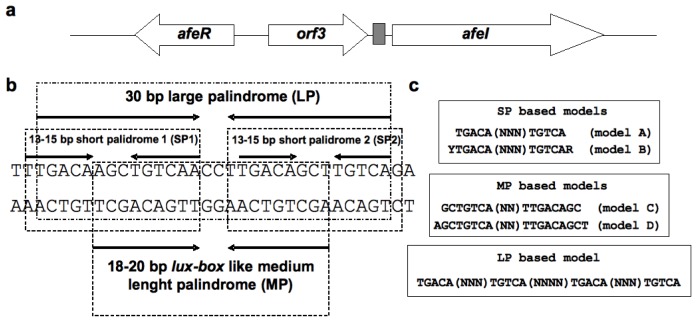
Generation of HMM from hypothetical alignments based on the *afe-box* sequence. (**a**) QS locus of *At. ferrooxidans* ATCC 23270. The gray colored rectangle indicates the location of the *afe-box* site; (**b**) Palindromic structures identified in the *afe-box* site. Palindromes are indicated by arrows. SP1 and SP2 sequences are inverted repeats of each other; (**c**) Representation of the alignments performed to generate the different HMM.

The 30 bp palindromic sequence found in the intergenic region between *afeI* and *orf3* genes of the *At. ferrooxidans* QS locus (*afe-box*) has an unexpected sequence complexity (Figure 1). Excepting the two central nucleotides, it has nearly perfect dyad symmetry. The 30 bp large palindrome (LP) can be subdivided into two shorter palindromic structures comprising each half of the sequence (SP1 and SP2) where these two sequences are inverted repeats of each other. The *lux-box* consensus sequence previously described [13] is located within the central 20 base pairs and is denoted “MP” after “medium length palindrome”. Thus, based on conservation of palindromic structure and symmetry (see materials and methods), different HMMs were constructed: HHM-models “A” and “B” from SP sequences, “C” and “D” from the MP sequence, and “E” from the LP sequence (Figure 1c). All these models were used to screen the whole genomic sequence of *At. ferrooxidans* ATCC 23270.

### 2.2. Prediction of *AfeR* Binding Sites in the *At. ferrooxidans* ATCC 23270 Genome

Predicted binding sites (PBSs) were defined as “a fragment of the genome sequence where one or more HMM hit is found in either strand”. Thus, a total of 273 PBSs were identified. As expected, the A and B type PBSs were mainly overlapped. These two kinds of PBS were grouped in the “SP” set. The same kind of overlap occurred with the C and D HMM-models and therefore, these two kinds of PBSs were grouped in the “MP” set. The third set comprises the E model PBSs and thus it was named “LP”. Overlapping between the three kind of PBS sets was infrequent. Only five double sites (1 SP∩MP plus 2 MP∩LP plus 2 LP∩SP) and 2 triple sites (SP∩MP∩LP) were found. These overlapping sites were merged and each merge was considered a single PBS. Finally, the complete operation yielded a total of 265 unique PBSs.

**Figure 2:**
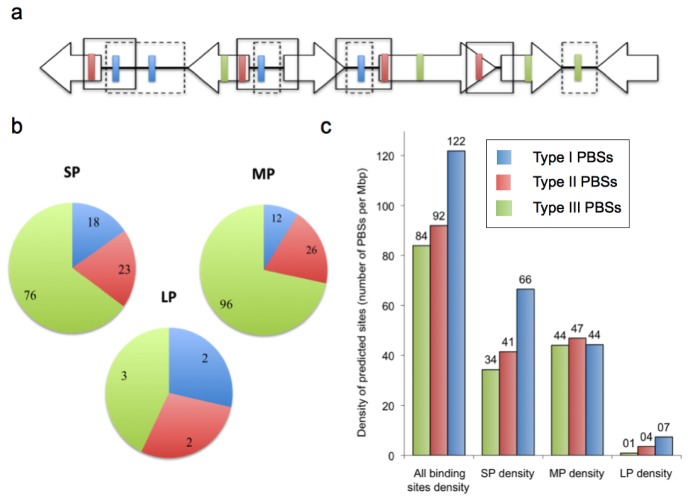
Distribution of predicted binding sites in the *At. ferrooxidans* ATCC 23270 genome sequence. (**a**) Hypothetical gene organization in the *At. ferrooxidans* genome showing the classification of predicted binding sites (PBSs) in three sets according to their position relative to an open reading frame start. PBSs found in sense intergenic regions (Type I) (see text) are indicated with blue rectangular blocks. Type II and Type III PBS are denoted with red and green, respectively. Intergenic regions are indicated by dashed squares. The continuous line denotes the permitted “250 bp zone” (see text); (**b**) Relative abundance of Type I, II and III PBSs in the total set; (**c**) Compared abundance of the SP, MP and LP in the three PBS types according to a calculated “density” parameter. Densities were calculated dividing the number of PBSs in each subset by the number of total bases in the *At. ferrooxidans* genome available for each subset (total intergenic bp in the genome (tIG) for Type I; sum of total “250 bp zones” (t250Z) minus tIG bp in the genome for Type II; total bp minus the sum of t250Z in the genome for type III).

The 265 PBSs were further classified in three different sets according to their proximity to a predicted start codon (Figure 2). The Type I set consists of the PBSs located in intergenic (IG) regions or overlapping an IG region that has a predicted start codon in their proximity, namely “sense” IG regions (*i.e.* the DNA region between the stop codons of converging genes is not a “sense” IG region). The Type II set consists of the PBSs that are found 200 bp upstream and 50 bp downstream from a predicted start codon and are not part of an IG region. Type III set consists on all PBS that are not part of Type II or Type III sets (Figure 2a). Approximately two thirds of the PBSs were part of the type III set. The rest of the PBS were nearly equally distributed between Type I and Type II sets (Figure 2b). An arbitrary density parameter, (*i.e.*, PBSs per megabase of possible DNA regions in each subset) was calculated to determine if the binding site density was homogeneously distributed along the whole sequence or if it was biased towards IG regions. Figure 2c shows that the density of PBSs is higher in “sense” IG regions (type I) than in other regions. This highest density is largely due to the bias created by the SP based PBSs, and not MP based PBSs. On the other hand, double and triple sites were excluded from this analysis because of the low number of occurrences.

### 2.3. Identification of Putative Gene Functions Regulated by *AfeR*

All Type I PBSs were selected as possible AfeR binding sites. PBSs belonging to the Type II set were selected only if their location almost overlapped or overlapped with a consensus sigma 70 (see Experimental Section). All PBSs belonging to the Type III set were discarded as probable binding sites due to their location. Applying this filter yielded a total of 62 PBSs (Table S1). Moreover, to improve the reliability of the PBS assignment, sequences of all selected PBSs plus 80 bp flanking each side were used to screen for common sequence motifs using the MEME motif discovery tool [17]. MEME searches for common motifs in a set of nucleotide or amino acid sequences. We applied the MEME tool without using the *afeI* upstream palindromic sequence as an input for this search. The results show that the most frequent and highest scoring motif was an 18-mer that corresponds to the MP based models present in each input sequence. The MEME program found the 18 bp motif in most of the MP-containing input sequences. On the other hand, the SP based PBSs were not detected as a MEME common motif. Interestingly, some of the SP containing input sequences possessed the MEME 18 bp motif near or overlapping the corresponding SP sequence. These new motifs were not detected as MP-based PBSs in the previous HMM search. Nevertheless, the MEME tool mainly confirmed the results obtained with the HMMs. Then, the gene functions associated with all these identified PBs were noted (Table S1). The identified gene functions whose expression could be controlled by AfeR were diverse, and there was not a clear biasing towards a particular functional category.

**Figure 3:**
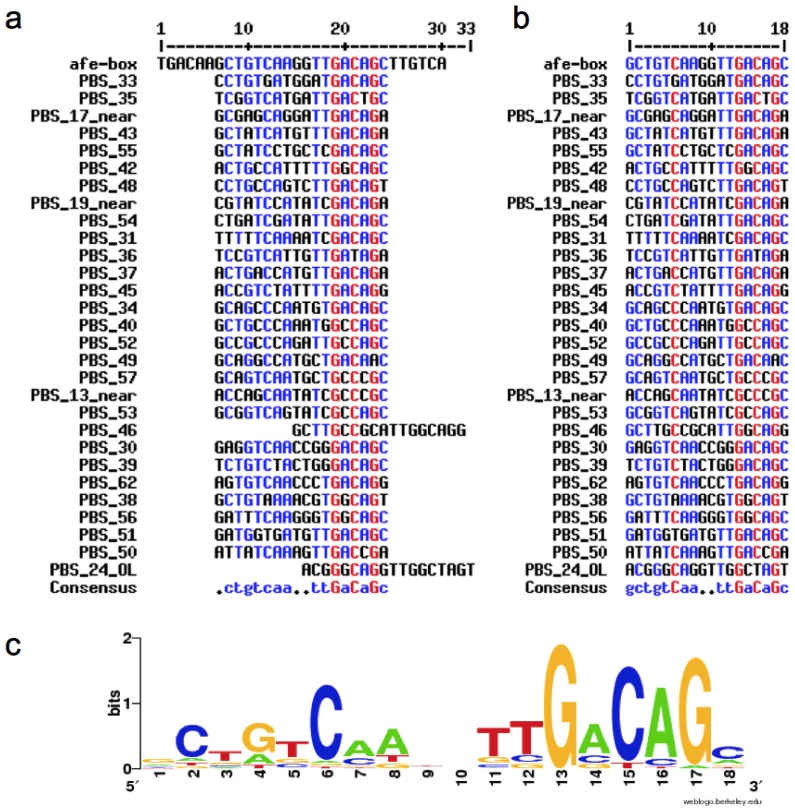
Multiple sequence alignment of selected predicted binding sites (PBSs) that were also positive for the MEME common motif search. Only MEME motifs located near (“_near”) or overlapping (“_OL”) SP type PBSs are indicated. (**a**) Alignment including the complete version of the afe-box (30 nucleotides); (**b**) Alignment including the central palindromic module of the afe-box (18 nucleotides). PBS_24_OL and PBS_46 align with better scoring to the right half of the complete afe-box. Numbers correspond to PBSs listed in Supplementary Table 1; (**c**) Sequence logo file created from the sequence alignment shown in b.

**Figure 4:**
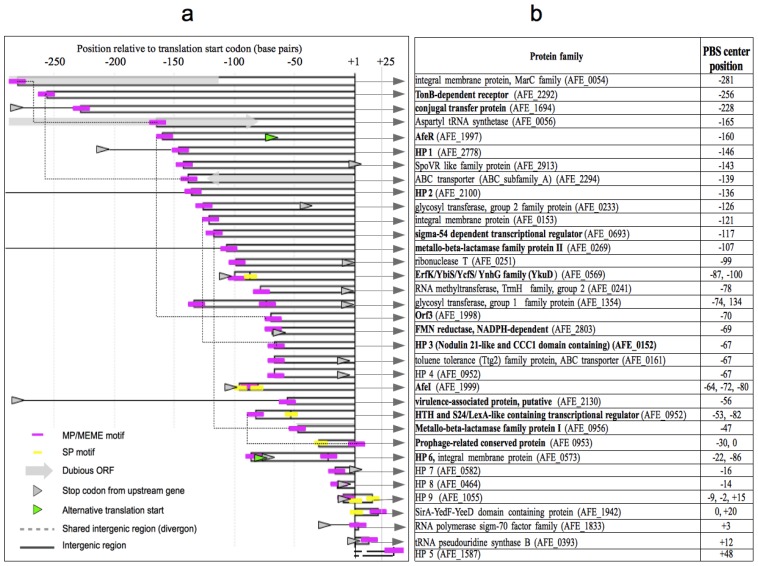
Genomic organization of selected predicted binding sites (PBSs) in the context of their putative gene targets. (**a**) Position of PBSs respect to a predicted translation start nucleotide. “+1” represents the position of the adenine in the start codon. The bars represent the distance between the start codon and the center of a particular PBS. Magenta rectangles and yellow rectangles indicate MP/MEME-type and SP-type PBSs, respectively. When more than one PBS is present upstream of the same gene, the rectangle is fragmented at that particular position and the corresponding square is centered there. Shared PBSs from diverging genes are connected by a dotted line. Stop codons of ORFs located upstream of the gene is indicated by a grey arrowhead. Absence of an arrowhead means that there is no upstream gene in the shown intergenic region range. Alternative start codons are indicated by a green arrowhead; (**b**) Identified gene functions downstream of the PBS-containing regions shown in “a” and PBS centers positions are indicated. Genes which have a PBS located in an intergenic region with no overlapping coding sequence, and that resemble the position of the afe-box are shown in boldface. The “AFE” gene number is indicated. Hypothetical proteins (HP) were numbered arbitrarily. To gain extra insight into the gene functions, current annotations where revised. For this reason, a slightly different annotation compared to the available one at CMR may appear.

Selected PBSs that also shared the MEME 18 bp common motif, including the new ones located in the SP-containing inputs, were aligned and a consensus sequence was generated using MultAlin [18] (Figure 3). The output from MEME provides a position-specific scoring matrix (PSSM) for the predicted motif. The PSSM generated for the 18 bp MEM motif was used as an input for the MAST program (Motif Alignment and Search Tool), which was applied to a database consisting of all intergenic regions in the genome [19]. Eight motifs with e-values lower than 10 were found where the lowest e-value corresponded to the *afeI* palindrome. Two out of eight motifs were located in intergenic regions between converging ORFs. Two previously identified motifs were those located upstream the *afeR* gene and AFE_2100 (annotated as a virulence-associated protein). One new motif was found in the intergenic region upstream the AFE_1504 ORF (annotated as “nifW protein”). Also a new motif was located between the diverging ORFs AFE_2800 and AFE_2799, which are a glyoxalase family protein and an Ada family-DNA-3-methyladenine glycosylase II transcriptional regulator, respectively. The positions of PBSs that where found using the MEME algorithm are summarized in Figure 4.

To evaluate the effectiveness of an alternative *lux-box* consensus-based search, the upstream 250 nucleotides from the four most identical sequences to the product of *afeI* in the genbank database (*luxI*-like genes from *Burkholderia pseudomallei* K96243, *Burkholderia cepacia* AMMD, *Burkholderia thailandensis* E264, *Burkholderia ambifaria* MC40-6) were used as input for the MEME discovery tool (the *afeI* palindrome containing upstream nucleotides was also used). In all four sequences a 18 bp motif that corresponded to a palindromic sequence similar to the *afe-box* was found (Results not shown). The PSSM generated was put into the MAST tool and the intergenic region database from *At. ferrooxidans* was screened for common motifs. The result from this procedure showed that only the *afeI* 18-bp motif was found. On the contrary, when the same database was screened using the PSSM generated from the MEME analysis of the MP-based PBSs, the number of hits was broader (see results). This suggests that using *lux-box* like sequences for the generation of HMMs in the *At. ferrooxidans* search would have yielded fewer hits, and hence a narrower search.

### 2.4. The *At. ferrooxidans* Putative Quorum Sensing Regulon

Functions regulated by QS in *At. ferrooxidans* are unknown. Here, we report results obtained from a bioinformatic approach to identify possible binding sites of the AfeR protein in the *At. ferrooxidans* ATCC 23270 genome sequence with the aim of defining a putative QS regulon for this bacterium.

HMMs were constructed based on alignments of hypothetical sequences that took the palindromic structure located upstream of the *afeI* gene as a template. Alignments of known *lux-box* type sequences to generate the HMMs were not performed because of the little overall conservation of these sequences [20]. The HMMs were designed to conserve palindromic zones and vary non-palindromic zones. This design allowed the retention of the modular nature of palindrome organization as opposed to sequence conservation only. Modularity may have a role in the mechanism of transcriptional regulation at the *afe-box* because different modes of binding are expected depending on the organization of sequence modules, as it has been experimentally tested for the *fur* binding site [21]. The fact that the canonical *lux-box* is only a fraction of a larger structure suggests that non-AfeR transcriptional regulators might also bind to the *afeI* operator.

The search yielded a total of 265 PBSs. SP model-based PBSs were preferentially located on intergenic regions whereas MP model-based PBSs were not (Figure 2). This can be interpreted as a “preference” of the SP model-based PBSs towards intergenic regions, hence raising the possibility that SP PBSs are true *cis-*acting regulatory motifs. Also, it is possible that this preference is due to the overlapping of the -35 element of a sigma 70 promoter from *afeI* with the modeled SP structure, thus implying that at least some of the SP hits may actually be -35 elements. Supporting the hypothesis mentioned above, the Bprom predictor found some Type I SP hits overlapping -35 elements but it also found hits overlapping -10 elements and hits with no overlapping at all. On the other hand, only MP model-based PBSs were found as common motifs when all PBSs were searched with the MEME tool, making MP PBSs more likely to be the correct regulatory motif for AfeR. Thus, as SP and MP based models did not show significant overlapping in the HMM search, the determinants for the AfeR interaction with DNA are likely to be enclosed within the central 18–20 nucleotides of the large 30 bp palindrome and that the SP PBSs may be binding sites for a distinct regulator. It is possible then that SP based PBSs may be part of a distinct regulon that overlaps the AfeR regulon in the transcriptional regulation of *afeI*. There are examples of multiple regulatory *cis-*acting elements upstream of various QS-controlled genes, including the regulatory zones of I and R genes [22,23]. These regulatory sequences can overlap *lux-box* like elements as is the case of the binding site for the regulator RsaL, upstream of *lasI* from *P. aeruginosa* [24] or the MetR (LysR-type transcriptional regulator) and CRP (catabolite repressor protein) binding sites upstream of *luxI* gene from *V. fischeri* [23].

Among the ORFs presented in the PBS table (Supplementary Table 1), five of them had two PBSs and one had three PBSs upstream of their predicted ATG start codon. Hypothetical protein AFE_1942, the diverging genes AFE_0999 (conserved domain protein) and AFE_0998 (putative transcriptional regulator), and the ErfK-YbiS-YcfS-YnhG family protein AFE_0569 had a SP model-based PBSs and a predicted MEME 18-bp motif. In the case of AFE_0569, the sequences are overlapped (Supplementary Table 1). AFE_1354 (a group 1 glycosyl transferase family protein) has 2 MP-based PBSs upstream from its start codon. Hypothetical protein AFE_1055 has two SP-based PBSs and a MEME 18-bp motif overlapping one of these. Examples exist where more than one *lux-box* like sequence is found upstream of QS-controlled genes. This is also the case for the *lasB*, *hcn* and *pqsA* genes from *P. aeruginosa* [25–27].

The type of regulation exerted over the AfeR targets remains unknown. AfeR can activate transcription from the *afeI* promoter in the presence of AHL [13], but the binding dependence on AHL remains to be determined. The ORFs found to be putatively regulated by the AfeR protein in this study have their respective PBSs located in various positions relative to predicted σ^70^ promoters. Some of them overlap -35 elements, others overlap -10 elements and others do not overlap either. Previously described LuxR family activators of transcription like TraR, LasR and LuxR itself generally bind a *lux-box* like element centered in position -43 (overlapping the -35 element of the σ^70^ promoter) in the presence of AHL. Some transcriptional regulators of the LuxR family bind their target sequences in the absence of and self-dissociate in the presence of AHL, as is the case of EsaR of *Pantoea stewartii* or ExpR_Ecc_ of *Erwinia carotovora* [28,29]. Both mentioned regulators are repressors of transcription. In this case, the location of the *lux-box* like sequence overlaps the -10 element of the σ^70^ promoter. The mentioned regulators can also function as activators of transcription in artificial genetic constructs where the position of the *cis*-acting element is changed to a -35 element overlap (maintaining the dependence on absence of AHL substrate) [30]. This shows that the ability to interact with RNA polymerase and activate transcription remains intact, and that activation rather than repression of other genes in its native context can occur depending on the *lux-box* like element position. Moreover, it has been demonstrated that genuine LasR binding sites are located as far as 383 bp from the translation start codon (*phzA* gene) [31], thus making our screening somewhat conservative.

None of the PBSs found had similar palindromic complexity to neither the *afeI* upstream sequence nor perfect dyad symmetry. Dyad symmetry is not a prerequisite for the *lux-box* like elements to interact with their cognate regulator as demonstrated for various QS-controlled genes in *P. aeruginosa* [20].

The gene functions associated to the 18 bp MEME motif have been related to several biological processes (Figure 4). One of these is QS itself, represented by AfeR and AfeI proteins and possibly the product of the *orf3* gene. The position of the *afe-box* located upstream of o*rf3* and *afeR* overlaps predicted -35 elements in both cases, suggesting an activator role for AfeR at this locus. Other LuxR-like proteins are also subject of self-regulation, as is the case for the positive feedback loop experienced by PhzR from *Pseudomonas fluorescens* [32] and the negative feedback loop involving EsaR from *Pantoea stewartii* [29].

There were two other transcriptional regulators forming part of the predicted targets: first, a helix-turn-helix transcriptional regulator that also contains a LexA motif and second, a Sigma 54-dependent transcriptional regulator. This is consistent with the fact that AfeR is a global regulator of transcription that controls the expression of many genes indirectly. The S24/LexA-like domain is related to the bacterial SOS response; specifically it catalyzes its own proteolysis separating the DNA-binding domain and the rest of the protein. (CD number: cd06529) The Sigma 54-dependent transcriptional regulator has also an HTH DNA-binding motif and also an AAA-type ATPase domain (CD number: cd00009).

In the same locus as sigma 54-dependent transcriptional regulator, but in the opposite orientation, there is a metallo-β-lactamase protein family gene. Interestingly, there is the second metallo-β-lactamase among the hypothetical regulon. These proteins belong to the same superfamily as the “quorum quenching” lactonase enzymes AiiA and AttM, frequently found in Gram-positive and Gram-negative bacteria, respectively [33]. Quorum quenching enzymes are capable of hydrolyzing the lactone ring of AHL molecules.

Another target related to β-lactam antibiotics is a protein that contains an ErfK/YbiS/YcfS/YnhG domain (also known as YkuD domain) which codes for a transpeptidase that functions as an alternative pathway for peptidoglycan cross-linking. This pathway provides resistance to β-lactam antibiotics because it functionally replaces the sensitive penicillin-binding protein (PBP).

We found four transport-related putative targets. The first two are the divergently organized genes encoding a TonB-dependent receptor and an ABC transporter (of the ABC_DR_subfamily_A). The TonB-dependent receptor is involved in the specific transport of substances across the membrane, of which iron-carrying siderophores are the best characterized ones (Conserved domain database (CD) number: cd01347). Nevertheless, a role in QS has been demonstrated for TonB in *Pseudomonas aeruginosa.* There, TonB mutants show an impairment in AHL production independently of its iron transport activity [34]. On the other hand, the extensively studied ABC transporter protein family is involved in similar processes including siderophore, drug, bacteriocin, glycoconjugate and peptide efflux (CD number: cd03230). A third transport-related protein is another ABC transporter present in the set is a “toluene tolerance protein” (Ttg2 family). However, it is unlikely that toluene itself is the substrate for this transporter (CD number: cl01074). Finally, the MarC-family protein spans the membrane several times and is also thought to convey multiple antibiotic resistance, however its precise activity is unknown (CD number: cl000919).

Five genes related to RNA are also predicted targets. The first one is a TrmH-family protein, which is known to methylate rRNA. rRNA methylation in bacteria strongly correlates with resistance to ribosome-targeted antibiotics [35]. A second activity related with RNA modification is a tRNA psudouridine synthase (TruB in *E. coli*). TruB catalyzes the isomerization of specific uridines in tRNA. The third RNA-related gene is the aspartyl-tRNA synthase. Finally, the RibonucleaseT (RNAseT) protein in *E. coli* RNAseT is involved in tRNA turnover. It has a 3′-5′exonuclease activity, also related with DNA polymerase III. RNAseT is only found in gamma-proteobacteria (CD number: cd06134).

Several proteins with diverse activities where found to be part of this putative regulon. First, a conjugal transfer protein (TrbF), thought to be part of the pilus required for the transfer of DNA. DNA transfer is a major QS-regulated phenotype in *Agrobacterium tumefaciens* [36]. The rest of the genes are a NADPH-dependent FMN reductase, a Prophage-related conserved protein and several hypothetical proteins.

Finally, two glycosyl-transferase activities were found among the putative regulon. These activities are usually related to polysaccharide biosynthesis. The two genes belong to different groups. The “Group 1” glycosyl-transferase protein transfer activated nucleotide-sugar molecules to several substrates, including protein, lipids and other sugar residues. The “Group 2” glycosyl transferase has the same activity but structurally it belongs to a different family (CD number: cd04186). It is possible that these proteins play a role in the biosynthesis of the exopolymeric substances (EPS) or the lipopolysaccharide. In addition, *zwf* a gene encoding for glucose 6-phosphate-1-dehydrogenase (G6PDH) has been also identified as part of this regulon. G6PDH is part of the pentose phosphate pathway and is directly related to the cellular pool of glucose 6-phosphate that is involved in the biosynthesis of EPS precursors UDP-glucose and UDP galactose [37]. On the other hand, mutation in *zwf* leads to an approximately 90% reduction in alginate, one of the EPS produced by *Pseudomonas aeruginosa* [38]. It is well established in several bacterial models that EPS synthesis is regulated by quorum sensing [8]. On the other hand, several studies have established that *At. ferrooxidans* is capable of synthesizing the EPS precursors UDP-glucose and UDP-galactose [39] and EPS is involved in the adhesion to solid surfaces by this microorganism [40,41]. Moreover, qRT-experiments indicated recently that transcription levels of *zwf* and *afeI* are increased in the presence of a tetrazolic AHL-analogue [42,43] suggesting that QS and EPS synthesis might be also connected in this microorganism.

## 3. Experimental Section

### 3.1. Construction of Hidden Markov Models (HMMs)

Fractions of the 30 bp palindrome located in the intergenic region between the *afeI* gene and the *orf3* open reading frame were used to generate HMMs. First, four defined-length palindromes were chosen (a 13-bp short palindrome, an 18-bp medium-length palindrome, a 20-bp medium-length palindromes and a 30-bp large palindrome) (Figure 1). Second, a multiple alignment was done for each by replacing the non-palindromic nucleotides in the chosen sequence by random nucleotides in a combinatorial manner. In this way, the 13-bp palindrome (TGACA-NNN-TGTCA) alignment consisted of the 64 possible sequences that can be obtained by replacing the “N”-positions with every possible nucleotide. In a similar way, the 18-bp (GCTGTCAA-NN-TTGACAGC), the 20-bp (AGCTGTCAA-NN-TTGACAGCT) and the 30-bp palindrome (TGACA-AGC-TGTCA-NNNN-TGACA-GCT-TGTCA) sequences are a result of the alignment of 16, 16 and 256 sequences, respectively. Additionally, a 15-bp palindrome (Y-TGACA-(NNN)-TGTCA-R) HMM was built. In the 15-bp model, the sequences aligned differed from each other at the level of central and flanking nucleotides. In this case, the three central nucleotides (NNN) included were the 3-mers (AAA), (CCC), (GGG), or (TTT). The “Y” and “R” positions correspond to pyrimidines and purines, respectively, resulting in 256 sequences aligned. The alignments were performed using ClustalW [44]. HMMs were constructed from them using the HMMER package 1.8.4 programs [45]. Subsequently, command *hmmls* was used for finding multiple non-overlapping matching hits in an *At. ferrooxidans* genome sequence obtained from the J. Craig Venter Institute- Comprehensive Microbial Resource database (JCVI-CMR; http://cmr.jcvi.org/).

### 3.2. Analysis of the Genomic Contexts of the Putative *AfeR* Binding Sites

Available ORF sequences contain limited annotated information. For this reason, they were further annotated and organized in sets using ARTEMIS [46]. The context of each PBS was annotated ased on ORF predicted product homology with known proteins using BLASTp [47] and the conserved domain database (CDD) [48]. The start codons were inferred after performing multiple alignments of the BLASTp hits that shared highest identity with the respective query using the clustalW algorithm [44]. Sequence editing and formatting for further analysis was done with FeatureExtract [49], Sequence Manipulation Suite [50] and FaBox [51]. Consensus sigma 70 bacterial promoter -10 and -35 elements were inferred using the Bprom predictor (www.softberry.com) Type I and Type II PBS sequences with additional 100 bp flanking each side ware used as input for Bprom. All Type I PBSs were further characterized. On the other hand, Type II PBSs were selected for further characterization only if a Bprom prediction occurred within the Bprom input sequence. Shared motifs were identified using the MEME motif discovery tool [17,52]. The input for MEME was the set of selected PBSs sequences with additional 80 bp flanking each side. The MAST program [19] was used to search additional motifs in a database consisting of all intergenic regions in the *At. ferrooxidans* genome. The input for the MAST program was the position specific scoring matrix (PSSM) generated from the common motif sequences derived from MEME analysis. Multiple alignments of selected PBSs and consensus sequence (Figure 3) were constructed using MultAlin [18]. The sequence logo was obtained using WebLogo.

## 4. Conclusions

Our bioinformatic prediction allowed the identification of several gene functions associated with the presence of upstream putative quorum sensing *cis*-regulatory elements. Among this set of genes, the *afeR* response regulator and the divergently organized conserved unknown gene *orf3* were identified. For both genes, the *cis*-regulatory element overlaps the -35 promoter elements suggesting that auto-regulation of AfeR may take place, as it occurs in *Vibrio fischeri* and several other gram-negative bacteria.

The identification of genes involved in the biosynthesis of exopolysaccharides strongly indicates that the QS pathway of *At. ferrooxidans* might be involved in biofilm formation and directly correlates with the increase of adhesion to pyrite and sulphur by *At. ferrooxidans* obtained by the addition of AHL molecules [14]. Further experiments including high throughput strategies such as microarray analysis and RNA deep sequencing are required to precisely characterize the QS pathway of *At. ferrooxidans*. However, it has been previously described that *zwf* is activated by QS in *P. aeruginosa* [53]. Moreover, in addition to qRT-PCR results related to *zwf* gene [51], the bioinformatic strategy used in this work has been recently supported by a proteomic study that has identified five members of the predicted regulon as being induced (SpoVR, AFE_2532 hypothetical protein, NrdA) and repressed (MinE, SixA) in biofilms compared to planktonic cells [54]. This suggests that a connection of QS with high cell-density biofilms may be expected. Nevertheless, the present work provides the context for designing experiments that would allow the characterization of some genes putatively belonging to the *At. ferrooxidans* QS regulon.

## Supplementary Information
