# Supplementary material for: Bioinformatic Prediction of Gene Functions Regulated by Quorum Sensing in the Bioleaching Bacterium Acidithiobacillus ferrooxidans
Source: Int J Mol Sci. 2013 Aug 16;14(8):16901–16. doi: 10.3390/ijms140816901 (PMC3759942; doi:10.3390/ijms140816901)
Supplement: Supplementary file 1 [file ijms-14-16901-s001.pdf]

Supplementary Information

Table S1. Characterization of selected PBSs in terms of their genomic contexts.

PBS	HMM type	MEME motif [a]	Diverging ORFs	Bprom overlap [b]	Locus [c]	Gene number	Common Name [d]	Gene Symbol	Main role	Subrole
1	SP	NO	NO	US -35	AFE_3112	1	cytochrome c-type biogenesis protein ResB, putative	ND	Energy metabolism	Electron transport
2	SP	NO	NO	-35	AFE_3110	2	Ubiquinol-cytochrome c reductase, cytochrome b subunit	petB-1	Energy metabolism	Electron transport
3	SP	NO	NO	NP	AFE_0011	3	cell division topological specificity factor MinE	minE	Cellular processes	Cell division
4	SP	NO	NO	DS -10	AFE_2775	4	membrane protein	ND	Cell envelope	Other
5	SP	NO	YES	US -35	AFE_2532	5	HP [f]			
				DS -10	AFE_2531	6	transcriptional regulator, LysR family	ND	Regulatory functions	DNA interactions
6	SP	NO	YES	NP	AFE_2468	7	ISAfe4, tranposase orf1	ND	Mobile and extra-chromosomal element functions	Transposon functions
				-35	AFE_2467	8	acetyltransferase, GNAT family	ND	Unknown function	Enzymes of unknown specificity
7	SP	NO	NO	DS -10	AFE_2314	9	phosphohistidine phosphatase SixA	sixA	Regulatory functions	Protein interactions
8	SP	NO	NO	-10	AFE_2281	10	phosphonate metabolism protein PhnJ	phnJ	Central intermediary metabolism	Phosphorus compounds
9	SP	NO	YES	-35	AFE_2201	11	cytosol aminopeptidase	pepA	Protein fate	Degradation of proteins, peptides, and glycopeptides
				US -35	AFE_2200	12	YjgP/YjgQ family protein	ND	Unknown function	General

Table S1. *Cont.*

PBS	HMM type	MEME motif [a]	Diverging ORFs	Bprom overlap [b]	Locus [c]	Gene number	Common Name [d]	Gene Symbol	Main role	Subrole
10	SP	NO	NO	-10	NA[g]	13	HP			
11	SP	NO	YES	NP	NA	14	mcbC-like_oxidoreductase [CDD: cd02142]			
				NP	AFE_2098	15	HP			
12	SP	NO	NO	-10	AFE_2025	16	glucose-6-phosphate 1-dehydrogenase	zwf	Energy metabolism	Pentose phosphate pathway
13	SP	near	NO	NP	AFE_1942	17	HP			
14	SP	NO	NO	DS -10	AFE_1399	18	HP			
15	SP	NO	NO	NP	AFE_1376	19	transposon, orf2	ND	Mobile and extra-chromosomal element functions	Transposon functions
16	SP	NO	NO	DS -10	AFE_1247	20	HP			
17	SP	near	NO	DS -10	AFE_1055	21	HP			
18	SP	OL	NO	-10						
19	SP	near	YES	NP	AFE_0999	22	HP			
				US -35	AFE_0998	23	transcriptional regulator, putative	ND	Regulatory functions	DNA interactions
20	SP	NO	NO	NP	AFE_0934	24	alanyl-tRNA synthetase	alaS	Protein synthesis	tRNA aminoacylation
21	SP	NO	NO	NP	AFE_0766	25	HP			
22	SP	NO	YES	-10	AFE_0693	26	sigma-54 dependent transcriptional regulator	ND	Regulatory functions	DNA interactions
				-10	AFE_0692	27	oxidoreductase alpha (molybdopterin) subunit	ND	Unknown function	Enzymes of unknown specificity
23	SP	NO	NO	NP	AFE_0655	28	ABC transporter, ATP-binding protein	ND	Transport and binding proteins	Unknown substrate
24	SP	OL	NO	US -35	AFE_0569	29	ErfK/YbiS/YcfS/YnhG family	ND	Unknown function	General
25	SP	NO	NO	DS -10	AFE_0404	30	argininosuccinate synthase	argG	Amino acid biosynthesis	Glutamate family

Table S1. *Cont.*

PBS	HMM type	MEME motif [a]	Diverging ORFs	Bprom overlap [b]	Locus [c]	Gene number	Common Name [d]	Gene Symbol	Main role	Subrole
26	SP	NO	YES	US -35	AFE_0108	31	RND efflux system, outer membrane lipoprotein, NodT family	ND	Transport and binding proteins	Unknown substrate
				US -35	AFE_0107	32	DNA-binding response regulator	ND	Regulatory functions	DNA interactions
27	SP	NO	NO	NP	AFE_0105	33	metal ion transporter, NRAMP family	ND	Transport and binding proteins	Cations and iron carrying compounds
28	SP	NO	YES	US -35	AFE_3191	34	type III restriction-modification system, Res subunit	ND	DNA metabolism	Restriction/ modification
				-35	AFE_3190	35	HP			
29	SP	NO	NO	-10	AFE_3182	36	transposase, interruption-N	ND	Disrupted reading frame, Mobile and extra-chromosomal element functions	Transposon functions
30	MP	YES	NO	DS -10	AFE_2913	37	SpoVR like family protein	ND	Unknown function	General
31	MP	YES	NO	-35	AFE_2803	38	FMN reductase, NADPH-dependent	ND	Unknown function	Enzymes of unknown specificity
32	MP	NO	NO	-10	AFE_2789	39	HP			
33	MP	YES	NO	NP	AFE_2778	40	HP			
34	MP	YES	YES	-10	AFE_2294	41	ABC transporter, ATP-binding protein, authentic frameshift	ND	Transport and binding proteins	Unknown substrate
				NP	AFE_2292	42	TonB-dependent receptor	ND	Transport and binding proteins	Cations and iron carrying compounds
35	MP	YES	NO	NP	AFE_2130	43	virulence-associated protein, putative	ND	Cellular processes	Pathogenesis
36	MP	YES	NO	NP	AFE_2100	44	HP			

Table S1. *Cont.*

PBS	HMM type	MEME motif [a]	Diverging ORFs	Bprom overlap [b]	Locus [c]	Gene number	Common Name [d]	Gene Symbol	Main role	Subrole
37	MP	YES	NO	NP	AFE_1833	45	RNA polymerase sigma-70 factor family	ND	Transcription	Transcription factors
38	MP	YES	NO	DS -10	AFE_1694	46	transfer protein	ND	Cellular processes	Conjugation
39	MP	YES	NO	DS -10	AFE_1587	47	HP			
40	MP	YES	NO	DS -10	AFE_1354	48	glycosyl transferase, group 1 family protein	ND	Cell envelope	Biosynthesis and degradation of surface polysaccharides and lipo-polysaccharides
41	MP	NO	NO	-35						
42	MP	YES	YES	-35	AFE_0957	49	sigma-54 dependent transcriptional regulator	ND	Regulatory functions	DNA interactions
				US -35	AFE_0956	50	metallo-beta-lactamase family protein	ND	Unknown function	Enzymes of unknown specificity
43	MP	YES	NO	-10	AFE_0952	51	HP			
44	MP	NO	NO	-10	AFE_0658	52	transcription termination factor Rho	rho	Transcription	Transcription factors
45	MP	YES	NO	NP	AFE_0582	53	HP			
46	MP	near	NO	DS -10	AFE_0577	54	membrane protein, putative	ND	Cell envelope	Other
47	MP	NO	YES	NP	AFE_0545	55	thiol:disulfide interchange protein DsbD	dsbD	Energy metabolism, Protein fate	Electron transport, Protein folding and stabilization
				US -35	AFE_0544	56	conserved hypothetical protein	ND	Hypothetical proteins	Conserved
48	MP	YES	NO	-35	AFE_0464	57	HP			
49	MP	YES	NO	NP	AFE_0393	58	tRNA pseudouridine synthase B	truB	Protein synthesis	tRNA and rRNA base modification

Table S1. *Cont.*

PBS	HMM type	MEME motif [a]	Diverging ORFs	Bprom overlap [b]	Locus [c]	Gene number	Common Name [d]	Gene Symbol	Main role	Subrole
50	MP	YES	NO	-35	AFE_0269	59	metallo-beta-lactamase family protein	ND	Unknown function	Enzymes of unknown specificity
51	MP	YES	NO	NP	AFE_0251	60	ribonuclease T	rnt	Transcription	RNA processing
52	MP	YES	NO	DS -10	AFE_0241	61	RNA methyltransferase, TrmH family, group 2	ND	Protein synthesis	tRNA and rRNA base modification
53	MP	YES	NO	DS -10	AFE_0233	62	glycosyl transferase, group 2 family protein	ND	Cell envelope	Biosynthesis and degradation of surface polysaccharides and lipo-polysaccharides
54	MP	YES	NO	-10	AFE_0161	63	toluene tolerance protein, putative	ND	Cellular processes	Detoxification
55	MP	YES	YES	US -35	AFE_0153	64	integral membrane protein	ND	Cell envelope	Other
				-10	AFE_0152	65	HP			
56	MP	YES	NO	NP	AFE_0056	66	aspartyl-tRNA synthetase	aspS	Protein synthesis	tRNA aminoacylation
57	MP	YES	NO	US -35	AFE_3128	67	HP			
58	MP	NO	YES	-35	AFE_2638	68	ribonucleotide-diphosphate reductase, alpha subunit	nrdA	Purines, pyrimidines, nucleosides, and nucleotides	2'-Deoxy-ribonucleotide metabolism
				DS -10	AFE_2637	69	tldD protein	tldD	Regulatory functions	Other
59	MP	NO	NO	DS -10	AFE_1339	70	polysaccharide biosynthesis/export domain protein	ND	Cell envelope; Transport and binding proteins	Biosynthesis and degradation of surface polysaccharides and lipo-polysaccharide; Carbohydrates, organic alcohols, and acids

Table S1. *Cont.*

PBS	HMM type	MEME motif [a]	Diverging ORFs	Bprom overlap [b]	Locus [c]	Gene number	Common Name [d]	Gene Symbol	Main role	Subrole
60	MP	NO	YES	DS -10	AFE_0852	71	HP			
				-35	AFE_0851	72	translation elongation factor P	efp	Protein synthesis	Translation factors
61	MP	NO	NO	NP	AFE_0666	73	YCII-related domain protein	ND	Unknown function	General
62	MP-LP	YES	YES	-35	AFE_1998	74	HP			
				-35	AFE_1997	75	autoinducer-binding transcriptional regulator, LuxR family	ND	Regulatory functions	DNA interactions

[a] Selected PBSs sequences with additional flanking 80 bp regions in each side were used as inputs for the search of common motifs using the Multiple Em for Motif Elicitation (MEME) tool (Timothy *et al.* 1994). "NO" means that no motif was found by MEME. "YES" means that MEME identified the corresponding PBS as the common motif. "near" means that the common motif was found within the input sequence but it is not the corresponding PBS. "OL" means that the common motif overlaps the corresponding PBS but is not the same sequence. [b] Selected PBSs sequences with additional flanking 100 bp regions in each side were used as inputs for the search of putative σ^{70} consensus promoters using the Bprom prediction tool (www.softberry.com). "-35" means that PBS overlapped the predicted -35 element. "-10" means that PBS overlapped the predicted -10 element. "DS -10" means that the corresponding PBS was located downstream of the -10 element. "US -35" means that the corresponding PBS was located upstream of the -35 element. "NP" means that there was no prediction of σ^{70} promoter. [c] ORF identity assigned as in NCBI Reference Sequence: NC_011761.1. "NA" means that the ORF was not annotated as such in the list. [d] Gene product identity assigned by JCVI-CMR. "HP" means that the product is an hypothetical protein or a conserved hypothetical protein. [e] Gene symbol assigned by JCVI-CMR. "ND" means no data. [f] Hypothetical proteins do not have main or subrole assignations. [g] Non Applicable. These correspond to non-annotated ORFs. They were manually curated. If putative conserved domains were detected, the highest scored one is indicated. The coordinates of the ORFs are also indicated between parentheses.

© 2013 by the authors; licensee MDPI, Basel, Switzerland. This article is an open access article distributed under the terms and conditions of the Creative Commons Attribution license (http://creativecommons.org/licenses/by/3.0/).
